# Supplementary material for: Cardiac Magnetic Resonance to Detect the Underlying Substrate in Patients with Frequent Idiopathic Ventricular Arrhythmias
Source: Diagnostics (Basel). 2021 Jun 18;11(6):1109. doi: 10.3390/diagnostics11061109 (PMC8233842; doi:10.3390/diagnostics11061109)
Supplement: Supplementary file 1 [file diagnostics-11-01109-s001.zip › diagnostics-1264808-SI.pdf]

Supplementary Table S1. Baseline characteristics of the 72 patients included in the study.

**Supplementary Table 1. Baseline patient characteristics**	
Clinical characteristics	
Age (years)	47 ± 15
Males, n (%)	34 (47.2)
BSA (m^2)	1.9 ± 0.2
BMI (kg/m^2)	27.2 ± 4.9
Smoking, n (%)*	15 (24.2%)
Clinical indication	
PVCs, n (%)	56 (77.8%)
NSVT, n (%)	16 (22.2%)
PVCs on 24-h Holter, (n)	15936 ± 12894
PVC/total QRS complexes, %	14.7% ± 11.4%
Medical history	
Hypertension, n (%)†	12 (17.4%)
Dyslipidaemia, n (%)†	25 (36.2%)
Thyroid disease, n (%)†	9 (13.0%)
Diabetes	1.4%
Antiarrhythmic therapy	
Beta-blockers, n (%)‡	43 (63.2%)
Calcium antagonists, n (%)‡	1 (1.5%)
Sodium channel blockers, n (%)‡	7 (10.3%)
Amiodarone, n (%)‡	3 (4.4%)
None, n (%)‡	14 (20.6%)

BMI: body mass index; BSA: body surface area; NSVT: non-sustained ventricular tachycardia; PVC: premature ventricular contraction. *14% missingness; †4% missingness; ‡5.6% missingness.

Supplementary Table S2. Cardiac Magnetic Resonance findings in patients and healthy control subjects.

**Table 2. CMR findings**			
	**All patients (n= 72)**	**Controls (n= 72)**	***p* value**
LVEDV (ml)	162.0 [138.3, 178.3]	147.5 [128.5, 173.5]	**0.045**
LVEDV index (ml/m^2)	84.0 [75.4, 92.3]	79.0 [69.0, 87.3]	**0.012**
LVESV (ml)	62.5 [52.3, 80.5]	51.5 [43.8, 61.3]	**<0.001**
LVESV index (ml/m^2)	33.7 [28.3, 41.8]	27.0 [24.3, 31.5]	**<0.001**
LVSV (ml)	93.5 [80.5, 106.0]	95.0 [83.8, 109.0]	0.378
LVSV index (ml/m^2)	48.7 [44.2, 54.4]	50.9 [45.3, 56.8]	0.178
LV ejection fraction (%)	59.5 [56.0, 64.0]	65.0 [63.0, 67.0]	**<0.001**
LV mass (g)	101.5 [80.8, 117.0]	103.0 [87.8, 120.8]	0.298
LV mass index (g/m^2)	52.7 (9.7)	55.5 (9.4)	0.075
RVEDV (ml)	149.0 [122.5, 171.5]	144.5 [122.0, 172.3]	0.971
RVEDV index (ml/m^2)	76.9 [68.4, 86.0]	77.6 [67.0, 85.2]	0.837
RVESV (ml)	55.0 [43.5, 67.0]	49.5 [38.8, 59.3]	0.07
RVESV index (ml/m^2)	28.5 [23.7, 34.5]	25.6 [21.9, 30.0]	0.053
RVSV (ml)	93.0 [78.0, 103.0]	93.5 [85.8, 111.8]	0.159
RVSV index (ml/m^2)	28.5 [23.7, 34.5]	25.6 [21.9, 30.0]	0.053
RV ejection fraction (%)	62.6 ± 6.0	66.1 ± 4.3	**<0.001**

EDV: end-diastolic volume; ESV: end-systolic volume; LV: left ventricular; RV: right ventricular; SV: stroke volume. Values with normal distribution are shown as mean ± standard deviation, while values without normal distribution are shown as median with interquartile range.

Supplementary Table S3. Procainamide dose and effect on haemodynamic parameters and arrhythmia burden.

**Procainamide Administration**	**Before**	**After**	***p*-Value**
Mean procainamide dose (mg): 536 ± 200<br>Range (mg): 200 - 1000	-	-	-
Systolic blood pressure (mmHg)	131 ± 19	119 ± 18	<0.001
Diastolic blood pressure (mmHg)	68 ± 15	65 ± 13	0.132
Heart rate (bpm)	76 ± 12	75 ± 14	0.483
Image Quality<br>(1 bad, 2 moderate, 3 very good, 4 excellent)	1.52 ± 0.51	3.48 ± 0.63	<0.001
Result	No. of patients (%)		
Complete PVCs suppression	10 (34.5%)		
Significant PVCs reduction	18 (62.1%)		
No response	1 (3.4%)		

The average dose of procainamide administered i.v. on the scanner table prior to Cardiac Magnetic Resonance scanning was 536 ± 200 mg (range 200-1000 mg) with an average duration of administration of 11 ± 4 minutes. There was a small but statistically significant drop in systolic blood pressure after procainamide administration, but no significant drop in diastolic blood pressure or heart rate. An operator graded image quality on cine images before and after procainamide administration. A 1 to 4 scale system was used: 4 – no artefacts - excellent image quality; 3 – minor artefacts with overall good image quality; 2 – moderate artefacts affecting significantly image quality and the accuracy of the analysis; and 1 – major artefacts, scan not analysable at all. Overall image quality significantly improved after procainamide administration. Paired t-tests were used to compare blood pressure and heart rate response of the patients, and image quality before and after procainamide administration. All tests were two-sided and α was set at 0.05. PVCs: premature ventricular contractions.

Supplementary Table S4. Possible origin of ventricular arrhythmia based on ECG findings.

**Supplementary Table 4. Characteristics of ventricular arrhythmia**	
Origin of ventricular arrhythmia	Number of patients* (%)
RV outflow tract	40 (61.5%)
LV outflow tract / coronary sinus	5 (7.7%)
Mitral annulus / aortomitral continuity	4 (6.2%)
LV origin	11 (16.9%)
RV origin	2 (3.1%)
Two morphologies	3 (4.6%)

LV: left ventricular; RV: right ventricular. *9.7% missingness
